# High Kynurenine (a Tryptophan Metabolite) Predicts Remission in Patients with Major Depression to Add-on Treatment with Celecoxib

**DOI:** 10.3389/fpsyt.2017.00016

**Published:** 2017-02-13

**Authors:** Daniela Krause, Aye-Mu Myint, Christine Schuett, Richard Musil, Sandra Dehning, Anja Cerovecki, Michael Riedel, Volker Arolt, Markus J. Schwarz, Norbert Müller

**Affiliations:** ^1^Department of Psychiatry and Psychotherapy, Ludwig Maximilian University, Munich, Germany; ^2^Institute for Transfusion Medicine, Ernst Moritz Arndt University, Greifswald, Germany; ^3^Department of Psychiatry and Psychotherapy, Wilhelm University, Muenster, Germany; ^4^Institute of Laboratory Medicine, Ludwig Maximilian University, Munich, Germany

**Keywords:** depression, cyclooxygenase-2, celecoxib, kynurenines, remission

## Abstract

**Background:**

Signs of an inflammatory process have been described in major depression.

**Methods:**

In a double-blind, randomized study of celecoxib or placebo add-on to reboxetine in 40 depressed patients, celecoxib treatment has beneficial effects. In order to evaluate the tryptophan/kynurenine metabolism and to identify predictors for remission, tryptophan (TRP), kynurenine (KYN), kynurenic acid (KYNA), and quinolinic acid (QUIN) were estimated in the serum of 32 patients before and after treatment and in a group of 20 healthy controls.

**Results:**

KYN levels were significantly lower in patients (*p* = 0.008), and the QUIN/KYN ratios were significantly higher (*p* = 0.028). At baseline, the higher KYN/TRP ratio was predictive for remission during celecoxib add-on treatment (*p* = 0.04) as well as for remission in the overall patient group (*p* = 0.01). In the placebo group, remitters showed a higher KYNA/QUIN ratio (*p* = 0.032). In the overall group, remitters showed lower KYNA/KYN (*p* = 0.035) and QUIN/KYN (*p* = 0.011) ratios. The lower the formation of downstream metabolites, especially QUIN, the better the treatment outcome.

**Conclusion:**

The high KYN/TRP ratio predicted remission after treatment with celecoxib in this small sample of depressed patients. Eventually, the KYN/TRP ratio might be a marker for those patients, which benefit from an additional anti-inflammatory treatment.

## Introduction

Activation of the inflammatory response system in major depression (MD) is well documented (1–5). Recent meta-analyses clearly showed elevated interleukin-6 (IL-6) levels in patients with MD (6–9).

However, the findings of these meta-analyses differed regarding levels of the inflammatory markers C-reactive protein (CRP), IL-1, IL-1RA, and TNF-α, with more hints toward increased CRP levels and no association for TNF-α and IL-1 in depression (8). In general, the inflammatory response system appears to be activated, but the levels of the different markers vary across studies.

Prostaglandin E2 (PGE2) is an important mediator of inflammation (10). Increased PGE2 in the saliva, serum, and cerebrospinal fluid of depressed patients has been described previously (11–14). The enzyme cyclooxygenase-2 (COX-2) is involved in the function of PGE2 in the inflammatory pathway. The COX-2 inhibitor celecoxib, an add-on to different antidepressants, has demonstrated beneficial effects in the treatment of depression (15, 16). Although not all patients who received celecoxib add-on remitted, celecoxib showed significant advantages over the placebo add-on. However, side effects, including cardiovascular effects, have been observed during the use of COX-2 inhibitors, particularly in long-term treatment. With these specific side effects of celecoxib, screening and monitoring for cardiovascular risk factors and events is important, when treating MD with COX-2 inhibitors. Also, a recent meta-analysis with a total of 150 patients has shown that the adjunctive celecoxib group had better remission and response rates than the placebo group (17).

Taken this together, it would be desirable to predict remission to the therapy with celecoxib. Predictive markers of the immune system for antidepressant therapy response have been described before. Decreased IL-6 levels were predictive for response to antidepressant pharmacotherapy (18, 19). A very recent study identified increased cytotoxic T cells and decreased natural killer cells as possible predictors for treatment response in MD (20).

Additionally, a meta-analysis showed that persistently elevated TNFα was associated with prospectively determined treatment resistance for depressed patients (21). Products of the tryptophan/kynurenine metabolism, however, have not yet been studied under the aspect of antidepressant therapy, although they are induced by an enhanced inflammatory response and proposed to be involved in the pathophysiology of depression (22, 23). Enzymes of the tryptophan–kynurenine metabolism are regulated by pro-inflammatory cytokines and prostaglandin E2 as a coactivator, in particular the indoleamine 2,3-dioxygenase (IDO), which metabolizes tryptophan to kynurenine. Moreover, metabolites of tryptophan metabolism are plausible biomarkers for depression since the biological ranges are fairly narrow, the detection rate in blood is good, and they discriminate satisfactorily between depressed patients and controls (24). The precise degradation of tryptophan leads to different neurotransmitters that are excitotoxic (25) or *N*-methyl-d-aspartate receptor antagonists (26). The hypothesis of the current study is that the measurement of the way tryptophan is metabolized could help to identify remitters already before the onset of treatment. Therefore, in this 6-week study, we evaluated key tryptophan metabolites to investigate whether they predict the outcome of treatment with celecoxib as an add-on to an antidepressant.

## Materials and Methods

### Patients and Controls

In total, 60 subjects participated in this study. Of these, 40 participants were patients (20 males and 20 females) aged between 23 and 63 years. All patients were diagnosed with MD according to DSM IV (DSM IV: 296.2 × single depressive episode or 296.3 × recurrent depressive episode) and needed to have a 17-item Hamilton Depression Scale (HAMD-17) score of at least 15 (range for included depressed patients was from 15 to 38). Patients suffering from psychotic depression or also other inflammatory diseases (e.g., multiple sclerosis, rheumatoid arthritis, and inflammatory bowel disease) were excluded. Current intake of NSAID for any reason (including pain) was an exclusion criterion. Also, a history of substance or alcohol abuse/dependence and severe physical illnesses were exclusion criteria. Each patient was included after written informed consent. The study was examined and approved by the ethics committee of the medical faculty of the University of Munich in accordance with the Declaration of Helsinki 1975, revised Hong Kong 1989. The depressed patients were study participants of a double-blind randomized, placebo-controlled, and prospective parallel group trial of celecoxib add-on to reboxetine. After a wash-out period (or without, in case patients were not medicated) of 3–7 days (according to the prior drug used; no patient had prior fluoxetine treatment), the patients were randomized to either celecoxib or placebo. The treatment period lasted 42 days (6 weeks). Patients were permitted to take benzodiazepines if needed for bridging the gap until reboxetine showed its effects. The results of the clinical parameters were reported in detail elsewhere (16). Briefly, 20 of the 40 patients (12 males, 8 females) were allocated to treatment with celecoxib as an add-on to reboxetine and 20 (8 males, 12 females) to placebo add-on to reboxetine. The dose of reboxetine was flexible and ranged from 4 to 10 mg/day, according to the clinical needs. Celecoxib was administered at a dose of 400 mg/day.

The baseline mean scores on the HAMD-17 were 25.4 (SD 4.0) in the celecoxib group and 24.6 (SD 5.9) in the reboxetine plus placebo group. After exclusion of drop-outs and patients whose blood samples were no longer available from the baseline sample collection, a total of 32 subjects (18 in the celecoxib group and 14 in the placebo group; age 44.6 ± 11.6 years; age range: 25–65 years; 16 females, 16 males) were included in the study. Remitters were defined as patients whose scores on the HAMD-17 had decreased to 7 or less by the end of the study. Six of 18 patients in the celecoxib group and 3 of 14 in the placebo group were remitters. The relatively high drop-out rate (in particular in the reboxetine plus placebo group) might partly be explained by the limited antidepressant effects of reboxetine and partly by the side effects of reboxetine (16).

A total of 20 healthy, age-matched controls (age 40.0 ± 10.4 years; age range: 24–60 years; 5 females, 15 males) were recruited to allow comparison of the tryptophan metabolism parameters. A non-structured clinical interview was used to exclude participants with a personal or familial history of psychiatric illness, diagnosed autoimmune disease, or substance or alcohol abuse. These interviews were performed by an experienced psychiatrist. The healthy controls were free of chronic or acute physical illness associated with altered states of immunity and showed normal blood chemistry values (this included normal ranges of complete blood count, liver and renal function, and thyroid hormones). Please see Table 1 for characteristics of study participants.

**Table 1:** **Characteristics of study participants**.

Patient’s characteristics	Celecoxib group (*n* = 18)	Placebo group (*n* = 14)	Controls (*n* = 20)
Sex	11 males, 7 females	5 males, 9 females	15 males, 5 females
Mean age (years)	44.6 ± 11.5	43.9 ± 13.3	40.0 ± 10.4
Remitters	6 with HAMD-17 < 7	3 with HAMD-17 < 7	
Mean benzodiazepine dose	2.4 ± 3.0 mg/day	2.7 ± 3.1 mg/day	

The study was approved in accordance with the ethical standards of the responsible committee on human experimentation [medical faculty of the Ludwig Maximilian University (LMU)] or with the Declaration of Helsinki 1975, revised Hong Kong 1989. Written informed consent was obtained from each participant.

### Biochemical Analyses

Kynurenines were analyzed in serum obtained from fasting, early morning venous blood samples.

Tryptophan (TRP), kynurenine (KYN), and kynurenic acid (KYNA) were analyzed at the Psychoneuroimmunology Laboratory of the Department of Psychiatry and Psychotherapy, LMU, Munich, with high-performance liquid chromatography (HPLC) according to the method of Oades et al. (27). Analytes were extracted from the samples and calibrators/controls by using Waters Oasis MCX 1 cc (30 mg) extraction cartridges. The eluent was evaporated to dryness under nitrogen and reconstituted with 150 µl 0.1 M PBS. Reconstituted samples/calibrators/controls were analyzed with HPLC with 250 mm × 4 mm Supersphere 60 RP-select B, C8 column (Merck, Darmstadt, Germany). TRP (lex: 300 nm; lem: 350 nm) was measured by fluorescence detection, and KYN (365 nm) and KA (330 nm) were measured by UV detection.

Serum quinolinic acid (QUIN) was analyzed at the Laboratory of Immunology and Transfusion Medicine at the University of Greifswald on a Hewlett-Packard model 5988 quadrupole mass spectrometer operated in the electron capture negative chemical ionization mode with methane as the reagent gas (0.5 Torr). Sample extraction was performed according to Morrison et al. (28).

The coefficient of variation of the above analyses ranged from 7 to 10. Patient and control samples were analyzed in random order, and the technical assistants who analyzed the samples and read the chromatograms were blind to the diagnoses and treatment groups. Finally, in order to estimate TRP degradation, the KYN to TRP ratio (KYN/TRP) was calculated, which is an indirect marker for the activity of the enzyme indoleamine 2,3-dioxygenase. For the further degradation of KYN, the ratios KYNA/KYN and QUIN/KYN were calculated, as these ratios provide insights of the accumulation of neuroactive substrates.

### Statistical Analyses

Student’s *t*-test was used to compare normally distributed data between patients and controls and between different subgroups. Linear regression analysis was performed to analyze the effect of the parameters of TRP/KYN metabolism on remission to treatment (HAMD ≤ 7) in treatment subgroups, and multivariate analysis controlling for age and gender was performed to analyze the effect in the overall group. SPSS version 18.0 was used, and *p* < 0.05 was considered significant.

## Results

### Patients vs Controls

Serum KYN levels were significantly lower (1.78 ± 0.35 vs 2.04 ± 0.35 µg/ml; *t* = −2.78, *p* = 0.008) in patients (*n* = 32) than in controls (*n* = 20), but QUIN/KYN ratios were significantly higher (0.17 ± 0.05 vs 0.14 ± 0.03; *t* = −2.28, *p* = 0.028). Tryptophan did not differ significantly between the two groups. Serum KYNA levels showed a trend toward being lower in the patient group (0.311 ± 0.054 vs 0.347 ± 0.087 ng/ml; *t* = −1.808, *p* = 0.077).

### Celecoxib vs Placebo

Tryptophan metabolites did not differ significantly between the celecoxib (*n* = 18) and control groups (*n* = 14) at baseline or after 6 weeks of treatment.

### Remitters vs Non-Remitters of the Depressed Patients

**Figure 1:**
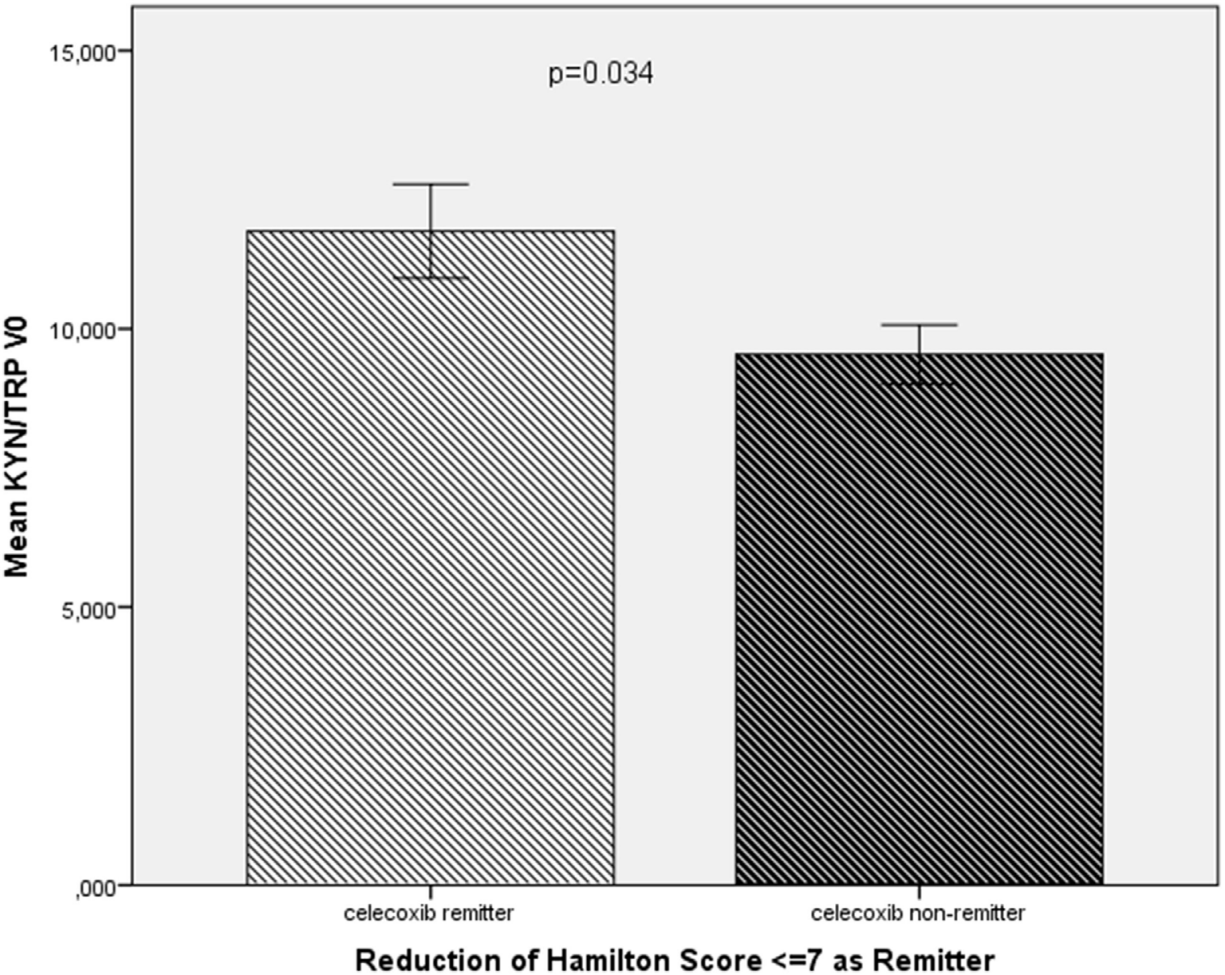
**Comparison of the KYN/TRP ratio at baseline (KYN/TRP V0) between remitters (*n* = 6) and non-remitters (*n* = 12) in the celecoxib add-on group**.

In the celecoxib group (add-on to reboxetine), remitters (*n* = 6) showed a higher KYN/TRP ratio (11.76 ± 2.07 vs 9.54 ± 1.68; *t* = 2.2, *p* = 0.034) at baseline (Figure 1). In the linear regression analysis, the baseline elevated KYN/TRP ratio was predictive for remission to celecoxib add-on treatment in terms of the percentage of patients showing a decrease in HAMD score to 7 or less at the end of the study (*B* = 0.03, CI = 0.001–0.059, *p* = 0.04).

In the placebo group (add-on to reboxetine), remitters (*n* = 3) showed a higher KYNA/QUIN ratio (1.36 ± 0.059 vs 1.07 ± 0.162; *t* = −2.25, *p* = 0.032).

**Figure 2:**
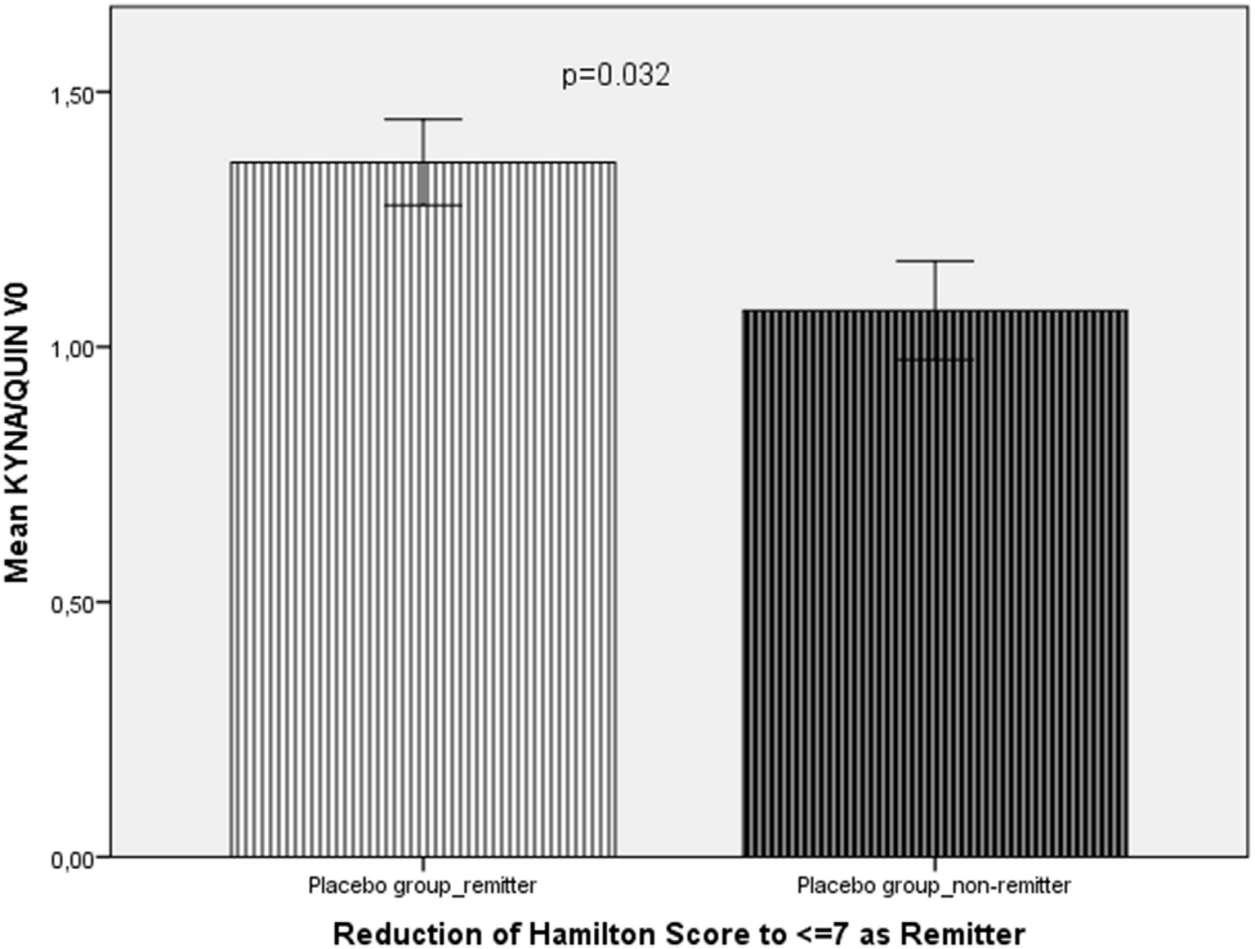
**Comparison of the KYNA/QUIN ratio at baseline (KYNA/QUIN V0) between remitters (*n* = 3) and non-remitters (*n* = 11) from the placebo group**.

**Figure 3:**
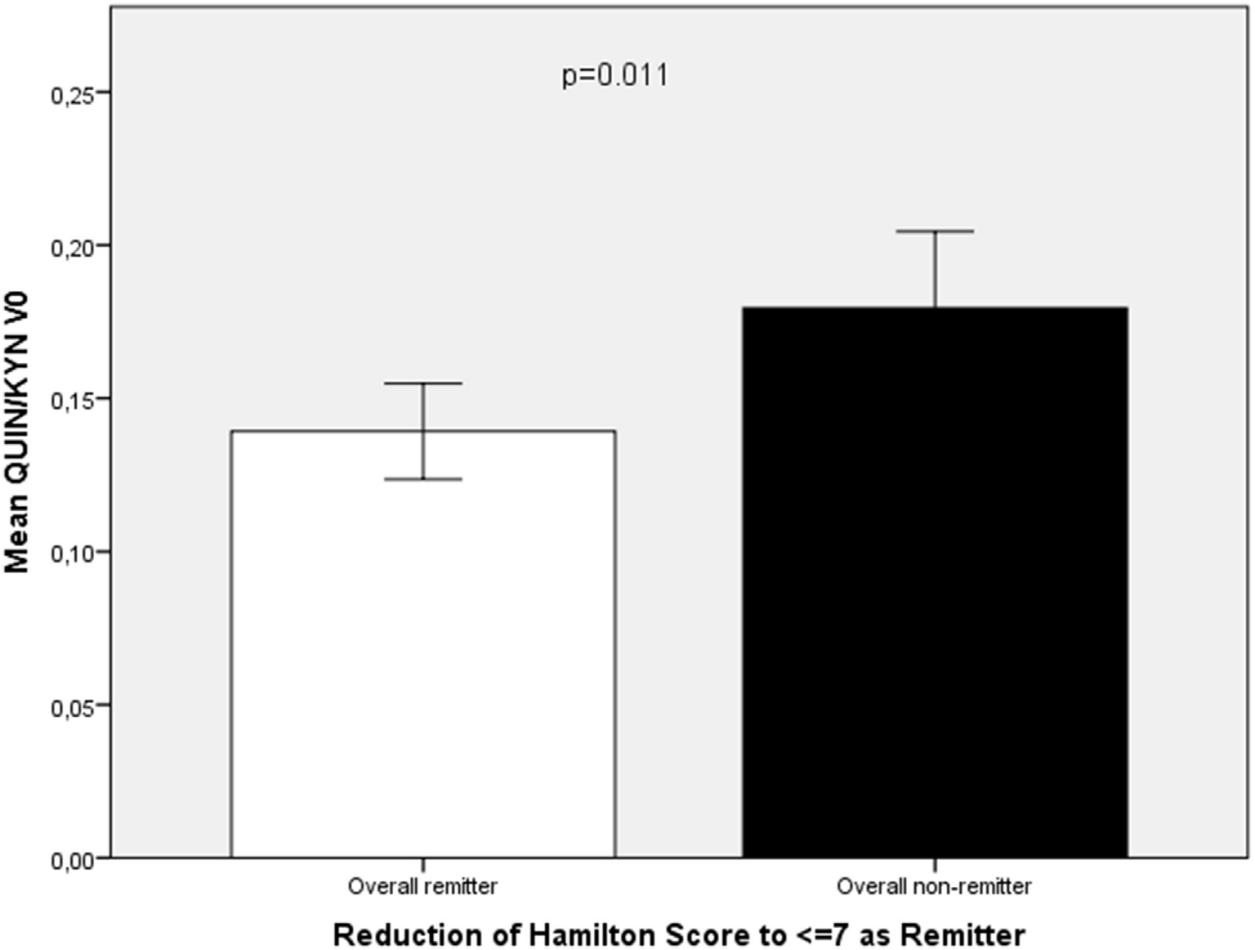
**Comparison of QUIN/KYN ratio at baseline (QUIN/KYN V0) between remitters (*n* = 9) and non-remitters (*n* = 23) from the overall group of patients with major depression**.

In the overall group (celecoxib and placebo add-on to reboxetine), remitters (*n* = 9) showed a higher KYN/TRP ratio (11.51 ± 1.81 vs 9.31 ± 1.99; *t* = −2.72, *p* = 0.011) (Figure 2) and lower KYNA/KYN (0.163 ± 0.017 vs 0.19 ± 0.051; *t* = −2.22, *p* = 0.035) (Figure 3) and lower QUIN/KYN (0.139 ± 0.022 vs 0.179 ± 0.057; *t* = −2.74, *p* = 0.011) ratios. When age and gender were controlled for, higher KYN/TRP was predictive for remission to antidepressant treatment with or without celecoxib add-on in terms of reduction of HAMD score to 7 or below (*B* = 33.012, *F* = 10.312, *p* = 0.004).

## Discussion

Our results show that initial serum kynurenine levels predict remission after add-on treatment with the COX-2 inhibitor celecoxib. More precisely, the higher KYN/TRP ratio at baseline predicted both remission in the celecoxib add-on group and in the whole (reboxetine) group. The KYN/TRP ratio indicates the activity of the enzymes tryptophan 2,3-dioxygenase (TDO) and indoleamine 2,3-dioxygenase (IDO), which reflect a response of the inflammatory system. Circulating monocytes have already been identified to overexpress inflammatory genes in depressed patients (6). Therefore, we conclude that the activity of the inflammatory response system predicts the outcome of treatment with the anti-inflammatory COX-2 inhibitor celecoxib: the greater the inflammatory response, the better the outcome after treatment with anti-inflammatory medication. However, a greater inflammatory response was associated also with better outcome after treatment with the antidepressant reboxetine alone. From a statistical point of view, it is relevant that two-thirds of the remitters were in the celecoxib group; therefore, the effect in the whole group is primarily due to the effect in the celecoxib group.

The ratios between the metabolites are intriguing (1) the ratio between KYNA—a downstream metabolite of KYN—and KYN (KYNA/KYN), and (2) between QUIN—another downstream metabolite of KYN—and KYN (QUIN/KYN) were significantly lower in the overall group of remitters. Also, the remitters in the placebo group showed a higher KYNA/QUIN ratio. QUIN is an NMDA-R agonist and excitotoxic (29), and KYNA is an NMDA-R antagonist (26). Overall, the results also suggest that the lower the formation of downstream metabolites, especially the excitotoxic QUIN, the better the treatment outcome. From the findings concerning the lower KYNA/KYN and lower QUIN/KYN ratios in remitters, it follows that the lower the degradation rate of KYN is to the downstream metabolites, the more KYN accumulates, and the higher the ratio is between KYN and TRP. A higher KYN/TRP ratio thus would mean less formation of downstream neurotoxic metabolites such as QUIN and subsequently would be associated with a better treatment outcome.

Our study shows that the KYN/TRP ratio may predict remission after antidepressant treatment with add-on anti-inflammatory medication using a COX-2 inhibitor, and this finding is also significant for the overall patient group. Our study also highlighted the fact that an increased formation of the downstream neuroactive KYN metabolites such as QUIN may negatively influence treatment outcome in depression. This finding is in line with the view that QUIN might be a pathogenetic factor in MD (30).

The increasing discussion of the role of inflammation and the increasing number of reports on beneficial effects of COX-2 inhibitors in MD demand the identification not only of biomarkers to characterize the immunopathology of MD but also of immune markers for treatment response and remission. Another study of celecoxib add-on reported that decreased serum levels of the pro-inflammatory cytokine IL-6 predict the response to treatment (31). Our findings and the IL-6 report strengthen not only the view that inflammation and the TRP/KYN metabolites play a role in the pathogenesis of MD but also that markers of this system may be suitable to predict treatment remission with anti-inflammatory compounds.

The present study also compared patients with MD to healthy individuals. We observed a higher QUIN/KYN ratio in the overall group of patients than in the healthy controls. This finding is in line with hypotheses previously proposed to explain the link between enhanced tryptophan degradation induced by activation of the inflammatory response system and subsequent enhanced formation of neurotoxic QUIN, thereby resulting in clinical symptoms such as depression (23, 32).

Our study has a major limitation: the overall sample size is rather small, thereby resulting in very small subgroups within each treatment group, e.g., of remitters and non-remitters. This small sample size has also some implications for statistics: no control for multiple comparisons has been performed. Therefore, the current results can only be interpreted with caution, and it is not yet possible to generalize them. Meaning, the present study should be seen as a pilot study for the identification of tryptophan metabolites as predictors for treatment remission and further studies with a larger sample size properly designed for a biomarker study should be performed. This future study could eventually divide patients into groups with high or low inflammatory markers. Nevertheless, our study can be seen as a first attempt on the way for a possible application of TRP/KYN metabolites to predict treatment outcome.

## Author Contributions

DK, NM, A-MM, MR, VA, and MS planned the study, wrote the study protocol, and prepared the manuscript. A-MM, MS, and MR calculated the statistics. MS, CS, and A-MM performed the lab work. DK, SD, and AC recruited the patients and samples and helped for interpretation of the data.

## Conflict of Interest Statement

The authors declare that the research was conducted in the absence of any commercial or financial relationships that could be construed as a potential conflict of interest.
